# Insight Into the Microbial Co-occurrence and Diversity of 73 Grapevine (*Vitis vinifera*) Crown Galls Collected Across the Northern Hemisphere

**DOI:** 10.3389/fmicb.2019.01896

**Published:** 2019-08-13

**Authors:** Han Ming Gan, Ernõ Szegedi, Rabeb Fersi, Samir Chebil, László Kovács, Akira Kawaguchi, André O. Hudson, Thomas J. Burr, Michael A. Savka

**Affiliations:** ^1^Deakin Genomics Centre, School of Life and Environmental Sciences, Deakin University, Geelong, VIC, Australia; ^2^Centre for Integrative Ecology, School of Life and Environmental Sciences, Deakin University, Geelong, VIC, Australia; ^3^School of Science, Monash University Malaysia, Bandar Sunway, Malaysia; ^4^National Agricultural Research and Innovation Centre, Research Institute for Viticulture and Enology, Kecskemét, Hungary; ^5^Laboratory of Plant Molecular Physiology, Center of Biotechnology of Borj Cédria, Hammam-Lif, Tunisia; ^6^Department of Biology, Missouri State University, Springfield, MO, United States; ^7^Western Region Agricultural Research Center, National Agricultural and Food Research Organization, Fukuyama, Japan; ^8^Thomas H. Gosnell School of Life Sciences, Rochester Institute of Technology, Rochester, NY, United States; ^9^Section of Plant Pathology, School of Integrative Plant Sciences, College of Agriculture and Life Sciences, Cornell University, Ithaca, NY, United States

**Keywords:** *Agrobacterium*, *Allorhizobium vitis*, amplicon sequence variants, crown gall disease, grape, microbiota, opine, vineyard

## Abstract

Crown gall (CG) is a globally distributed and economically important disease of grapevine and other important crop plants. The causal agent of CG is *Agrobacterium* or *Allorhizobium* strains that harbor a tumor-inducing plasmid (pTi). The microbial community within the CG tumor has not been widely elucidated and it is not known if certain members of this microbial community promote or inhibit CG. This study investigated the microbiotas of grapevine CG tumor tissues from seven infected vineyards located in Hungary, Japan, Tunisia, and the United States. Heavy co-amplification of grapevine chloroplast and mitochondrial ribosomal RNA genes was observed with the widely used Illumina V3–V4 16S rRNA gene primers, requiring the design of a new reverse primer to enrich for bacterial 16S rRNA from CG tumors. The operational taxonomic unit (OTU) clustering approach is not suitable for CG microbiota analysis as it collapsed several ecologically distinct *Agrobacterium* species into a single OTU due to low interspecies genetic divergence. The CG microbial community assemblages were significantly different across sampling sites (ANOSIM global *R* = 0.63, *p-*value = 0.001) with evidence of site-specific differentially abundant ASVs. The presence of *Allorhizobium vitis* in the CG microbiota is almost always accompanied by *Xanthomonas* and *Novosphingobium*, the latter may promote the spread of pTi plasmid by way of acyl-homoserine lactone signal production, whereas the former may take advantage of the presence of substrates associated with plant cell wall growth and repair. The technical and biological insights gained from this study will contribute to the understanding of complex interaction between the grapevine and its microbial community and may facilitate better management of CG disease in the future.

## Introduction

Plant-associated microbial communities are complex and diverse. As with most microbial communities, there is a limited understanding of the factors and mechanisms that establish and stabilize plant-associated microbiotas. It is unclear how specific populations of microorganisms are established and maintained and what promotes the appropriate balance of different microbes (Ramey et al., 2004). It is widely accepted that greater than 99% of the microbes present in many environments are not readily culturable or *not-yet-cultured* and therefore not easily accessible for basic and applied research (Bidle et al., 2007). The species diversity in many unique environments has never been described. To more fully understand novel environmental niches, several DNA-based methods have been developed including 16S rRNA gene analyses and metagenomics (Whitman et al., 1998; Williamson et al., 2005). The former provides information about taxa present in an environmental sample while the latter offers insight into the functional roles of different microbes within a community (Handelsman, 2004; Riesenfeld et al., 2004).

The grapevine-associated microbiota has been a subject of several studies due to the importance of grape cultivation for the production of wine, fresh grapes, raisins, jelly, juice, jam and grape seed extracts, and oil. The tissue saps of grapevines are rich in nutrients that include organic acids, amino acids, sugars, and several inorganic compounds with a pH 5.7–6.9 (Roubelakis-Angelakis and Kliewer, 1979; Andersen et al., 1989; Glad et al., 1992; Prima-Putra and Botton, 1998). This nutrient-rich tissue environment supports the growth of several bacteria resulting in an epiphytic and endophytic population with several species including important pathogens such as *Agrobacterium* spp. and *Allorhizobium vitis* (*A. vitis*) (Bell et al., 1995; Szegedi and Bottka, 2002; Bulgari et al., 2009; Compant et al., 2011). *Agrobacterium* spp. or *A. vitis* often causes galls to develop at the crown of the vine; hence, the name crown gall (CG), but can also induce galls on the perennial stems where wounds are inflicted as a result of grafting or injury by freezing temperatures or farm implements (Burr and Otten, 1999). Interestingly, the natural occurrence of CG on young green grapevine shoots has not been documented. CG tumors first appear in early summer as soft masses of disorganized cells which are creamy white or light green in color. In autumn, they become dry and wood-like and turn brown to black, hence the origin of the alternative name “black knot of grapevine” (Süle and Burr, 1998). Bark cracking and peeling may be associated with gall development. Profuse gall development may cause girdling of the trunk which prevents the exchange of nutrients between root and shoot systems thus leading to the reduced vigor of CG-affected vines.

The causal agent of CG tumor disease is commonly referred to as *Agrobacterium vitis* which was recently reclassified to the genus *Allorhizobium* based on whole genome phylogeny (Mousavi et al., 2014; Gan et al., 2018). Although less common, some strains of tumor-inducing (Ti) plasmid-harboring *Agrobacterium tumefaciens* can also cause CG (Pu and Goodman, 1992; Abdellatif et al., 2013). The Ti plasmid (pTi) encodes genes for the processing, transfer, and stable insertion of the transfer-DNA (T-DNA) from pTi to the plant nuclear genome. The constitutive expression of T-DNA oncogenes in the plant genome results in the overproduction of plant hormones cytokinin and auxin which causes the unregulated proliferation of undifferentiated plant cells which manifest themselves as the tumorous outgrowth of CG. Additional T-DNA-encoded genes produce enzymes that synthesize novel low molecular weight class of compounds known as opines (Bevan and Chilton, 1982; Chilton et al., 1982; Zambryski et al., 1989). The specific opine-type produced in the tumor is characteristic of the virulent *Agrobacterium*/*Allorhizobium* strain. Common opines found in CG include octopine, nopaline, and vitopine. These metabolites produced by the transformed plant tumor cells are almost exclusively metabolized as an energy source by the virulent agrobacteria that have induced the CG (Dessaux and Faure, 2018; Kuzmanović et al., 2018). A physiologically active CG expands the assortment of nutrients utilizable by bacteria and fosters a rich niche for plant-associated bacteria to colonize, grow, and form complex ecological interactions (Barton et al., 2018; Dessaux and Faure, 2018). In one study, 138 culturable bacterial colonies were isolated representing distinct morphological groups from eight grapevine CG tumors that produced octopine, nopaline, or vitopine (E. Szegedi, unpublished data). All isolates were non-fluorescent on King’s B medium indicating that none of them were fluorescent *Pseudomonas* species. On the basis of their morphological and physiological characters they could be allocated into three groups: (i) *A. vitis* type colonies (85), (ii) *A. tumefaciens* type colonies (8), and (iii) unidentified isolates which formed yellow colonies (45) (E. Szegedi, unpublished data). One of the unidentified yellow isolates (named Rr-2-17) was shown to accumulate large amounts of acyl-homoserine lactone quorum sensing signal molecules which can activate the *traR* promoter (used by the pTi for activation of pTi conjugation). This isolate was identified as a *Novosphingobium* sp. by full-length 16S rRNA gene sequencing and then verified via comparative genomics (Gan et al., 2009, 2012a). Further characterization of *Novosphingobium* sp. Rr2-17 showed the influence of the stringent response regulator, *rsh*, on the accumulation of the acyl-homoserine lactone quorum sensing signal (Gan et al., 2009).

Recent grapevine microbiota studies used Illumina 16S rRNA gene amplicon sequencing to investigate microbial communities associated with the grapevine organs such as leaf, fruit, cane, and root as well as soil surrounding the roots (Abdellatif et al., 2013; Pinto et al., 2014; Gilbert et al., 2014b; Belda et al., 2017; Manici et al., 2017; Morgan et al., 2017; Alaimo et al., 2018; Marasco et al., 2018; Wei et al., 2018). These studies provided intriguing insights into the effects of various environmental factors on the structure on the grapevine microbiota. Studies that focused on the microbiota of grapevine CG remain scarce with one of the first being conducted on samples collected from a single location in Germany across a temporal gradient (Faist et al., 2016). Using OTU clustering approach, Faist et al. (2016) showed that an OTU classified as *A. vitis* was the most common OTU in the CG affected graft unions followed by two OTUs belonging to *Enterobacter* and *Pseudomonas* with varying relative abundance from season to season. Recently, the amplicon sequence variant (ASV) approach is gradually gaining popularity as it can determine real biological sequences at single nucleotide resolution albeit at the expense of higher false positives (Callahan et al., 2017; Nearing et al., 2018). The CG microbiota is an excellent model to test the utility of this approach since members of the genus *Agrobacterium* are known to exhibit strikingly high interspecies 16S rRNA gene similarity (Gan and Savka, 2018).

In this study, we investigated the CG microbiota of grapevines from seven different vineyards located in Hungary, Japan, Tunisia, and the United States through Illumina amplicon sequencing of the 16S V3–V4 rRNA gene region. First, we implemented a new primer design to limit amplification of the grape chloroplast 16S rRNA gene, thereby substantially improving the representation of reads belonging to bacterial 16S rRNA. Second, we implemented an ASVs method to improve recovery of *Agrobacterium* 16S rRNA gene sequences with ecological implications by only removing noise from sequencing instead of clustering sequences based on similarity into operational taxonomic unit (OTU). Third, we provide evidence that CG tumors have a small core microbiota and that the microbiota structure is dependent on the sampling site and/or climate. Finally, we show that the abundance of *A. vitis* in the CG is positively correlated with the abundance of at least three non-*A. vitis* groups, e.g., *Novosphingobium* sp., *Xanthomonas* sp., and Microbacteriaceae sp., suggesting the presence of a microbial CG “hub” in the CG tumor environment.

## Materials and Methods

### Field Sampling and DNA Extraction

Crown gall tumor samples (1 tumor per grapevine) were collected from 73 grapevines in six vineyards from Hungary (two vineyards, *n* = 37), United States (two vineyards, *n* = 13), Tunisia (one vineyard, *n* = 21), Japan (one vineyard, *n* = 2) mostly in the summer or autumn (June–September) of 2013 and/or 2014 (see Supplementary Table S1 for sample-specific detail). In the northern hemisphere, CG tumors start to develop in late May (Jackson, 2014), therefore the age of the tumor tissue collected in this study ranges from approximately 1-month to 3-month-old. Each sample was collected by using separate sterile or flamed surgical blades and stored on ice during transportation to the lab. The DNA extraction was performed from approximately 200 mg of homogenized CG tissue within a day of sample collection using a modified CTAB method (Xu et al., 2005) or the Qiagen DNAeasy Plant Minikit (Qiagen) according to the manufacturer’s instructions (Supplementary Table S1). The extracted gDNA was sent to Monash University Malaysia for 16S amplicon library construction and Illumina sequencing.

### Amplification of the 16S V3–V4 Region and Illumina Sequencing

**FIGURE 1:**
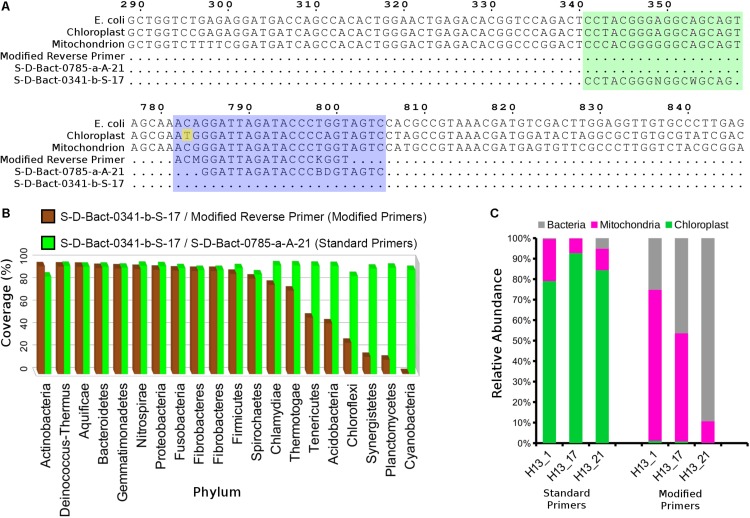
Modification of the 16S V3–V4 reverse primer reduced co-amplification of grapevine rRNA gene. **(A)** Nucleotide alignment of standard and modified primers against the grapevine mitochondrial and chloroplast rRNA genes. Green and purple blocks indicate 16S rRNA gene regions containing the aligned forward and reverse 16S V3–V4 primers, respectively. Numbers above the alignment indicate base position on the *Escherichia coli* 16S rRNA gene sequence. **(B)** Phylum-level taxonomic coverage of the standard and modified V3–V4 primer pairs as assessed by SILVA TestPrimer 1.0 based on the SILVA SSU r132 RefNR database (maximum number of mismatches = 5; length of 0-mismatch zone at 3′-end = 5 bases). **(C)** Relative abundance of bacterial, grapevine mitochondrial, and chloroplast sequence for three 2013 Hungarian crown gall samples that were amplified using the standard and modified 16S V3–V4 primers.

Single-step polymerase chain reaction (PCR) amplification was performed using NEBNext High-fidelity 2X PCR MasterMix (New England Biolabs, Ipswich, MA, United States) and Illumina adapter-containing primers (Caporaso et al., 2012) targeting the V3–V4 region (S-D-Bact-0341-b-S-17/S-D-Bact-0785-a-A-21) of the 16S rRNA gene (Klindworth et al., 2013). The cycling condition consisted of initial denaturation at 98°C for 1 min, 30 cycles of 98°C for 30 s, 58°C for 30 s, and 65°C for 1 min, followed by a final extension at 65°C for 5 min. However, due to heavy co-amplification of the chloroplast gene from an initial set of samples, a new reverse primer with a 3′-end base mismatch to the *V. vinifera* chloroplast sequence was designed to replace the reverse primer (Figure 1A). The phylum coverage of the reverse primer, when paired with the forward primer S-D-Bact-0341-b-S-17 was subsequently evaluated *in silico* using TestPrimer v1.0 (Klindworth et al., 2013). 16S rRNA amplicon from each sample was run on a 2% agarose gel and gel-extracted using E.Z.N.A. gel extraction kit (Omega Bio-Tek, Norcross, GA, United States). The gel-purified libraries were quantified using the KAPA library quantification kit Illumina (Kapa Biosystems, Cape Town, South Africa), normalized, pooled, denatured, and subsequently sequenced on a MiSeq Desktop Sequencer (Illumina, San Diego, CA, United States) located at Monash University Malaysia using the 2 × 250 bp run configuration.

### Bioinformatics Analysis

Polymerase chain reaction primer sequences were trimmed from the raw paired-end reads using Cutadapt v. 1.16 with the default setting. Reads that failed to be trimmed due to significant sequence mismatch were discarded. The adapter-trimmed paired-end reads were merged and filtered with fastq_mergepairs (default setting) and fastq_filter (-fastq_minlen 380 – fastq_maxee 0.25) implemented by Usearch v10.0.24010 to retain only ultra-high quality merged reads for the generation of ASV or OTU representative sequences (Edgar, 2010). The merged reads were combined into a single fasta file and labeled according to their sample ID using the add_qiime_labels.py script implemented in QIIME v1.9 (Caporaso et al., 2010) followed by singleton and doubleton removal using the “fastx_uniques” command in Usearch v10 (Edgar, 2010). Error correction (ASV approach) and OTU clustering of the dereplicated sequences used UNOISE3 and UPARSE, respectively (Edgar, 2013, 2016). The ASV/OTU table was constructed by mapping the unfiltered merged reads at 97% nucleotide identity threshold with Vsearch v2.8.0 to the ASV or OTU sequences (Rognes et al., 2016). Taxonomic assignment of the ASV/OTU was carried out in QIIME1 using RDP trained on the greengene v 13.8 database (DeSantis et al., 2006). Grapevine chloroplast- and mitochondrial-derived sequences initially identified by RDP naïve Bayesian Classifier (Wang et al., 2007) were validated by blastN search against their respective reference sequences on GenBank (NC_012119.1 and NC_007957.1) and removed from subsequent analysis.

### Microbiota Analysis

The chloroplast- and mitochondrion-filtered ASV table was rarefied to 28,364 sequences per sample and subsequently used to perform analysis of similarities (ANOSIMs) and to compute beta-diversity (Bray–Curtis distance matrix) and core microbiota in QIIME v1.9. For core microbiota computation, an ASV with >0.05% relative abundance is considered as “present” in a sample and the ASV needed to be present in at least 60% of the samples to be considered as part of the core microbiota. To identify ASVs that are significantly enriched in sites with more than five samples (biological replicates), we performed differential abundance analysis using the DESeq2 algorithm as implemented in the “differential_abundance.py” in QIIME v1.9 using the raw unrarefied OTU table (Supplementary Table S2) as the input.

### Microbial Association Network Construction

Co-occurrences were calculated with SparCC using unrarefied ASV table (Friedman and Alm, 2012). Pseudo *p-*values were calculated based on 100 bootstraps. Correlations were subsequently filtered based on statistical significance (*p*-value < 0.001), correlation coefficient strength (−0.5 < *R* < 0.5), and percentage of total observation count (>0.1%). Construction of the association network based on the filtered correlations was performed using Gephi v0.9.2 (Bastian et al., 2009).

### Phylogenetic Analysis

Amplicon sequence variants initially classified to the genus *Agrobacterium* by RDP and their corresponding exact OTU match were aligned with the 16S rRNA sequences of several described type strains belonging to the genera *Agrobacterium, Pararhizobium, Rhizobium*, and *Allorhizobium*. The alignment was performed using MAFFT v7.123b with the settings “–localpair –maxiterate 1000” (Katoh and Standley, 2013) and was subsequently trimmed with TrimAl v. 1.2 (Capella-Gutiérrez et al., 2009) retaining only the V3–V4 region to assess the accuracy of both RDP and 16S rRNA V3–V4 region in delimiting the genus *Agrobacterium*. Maximum-likelihood tree construction based on the trimmed alignment was performed using FastTree v2.1 and visualized in FigTree v1.4 (Price et al., 2010).

### Opine Detection and PCR Detection of Phytopathogenic *Allorhizobium* (*Agrobacterium*) *vitis* and *Agrobacterium tumefaciens* From Hungarian Crown Gall Samples

For all 16 Hungarian CG samples that were collected in 2014 (Supplementary Table S1 and Table 1), opine was extracted from approximately 100 mg of tumor tissue homogenized in distilled water as previously described (Szegedi, 2003). The extracted opine was separated with paper chromatography and stained using phenanthrene-quinone (for octopine and nopaline), silver-nitrate (for agropine and mannopine), or reversed silver nitrate (for vitopine) (Szegedi, 2003). PGF-PCR primers specific for the amplification of the polygalacturonase gene, *pehA*, from *A. vitis* were used to detect the presence of *A. vitis* in CG samples (Szegedi and Bottka, 2002). Detection of pathogenic *A. tumefaciens* strains used primers virD2A and virD2E which target the *virD* gene located on the Ti plasmid (Haas et al., 1995; Szegedi and Bottka, 2002). Purified gDNA of *A. vitis* strain Tm4 and *A. tumefaciens* A348 were used as positive controls for the PGF/PGR and VirD2A/VirD2E assays, respectively.

**TABLE 1:** Opine and molecular characterization of crown gall samples collected from Hungary in 2014.

**Sample ID**	**Cultivar**	**Opine assay**			**PCR assay**	
		**Phenantrene-quinone**	**Silver-nitrate**	**Reversed silver-nitrate**	**PGF-PGR**	**VirD2A-VirD2E**
H14_1	Lilla	Octopine	–	–	+	–
H14_2	Lilla	–	–	–	–	–
H14_3	Lilla	Octopine	–	–	–	–
H14_4	Lilla	Octopine	–	–	–	–
H14_5	Lilla	–	–	–	–	–
H14_6	Lilla	Octopine	–	–	–	–
H14_7	Medina	–	–	Vitopine	+	–
H14_8	Medina	–	–	Vitopine	–	–
H14_9	Medina	Octopine	–	–	–	–
H14_10	Teréz	–	–	–	–	–
H14_11	Teréz	Octopine	–	–	+	–
H14_12	Teréz	–	–	–	–	–
H14_13	Teréz	–	–	–	–	–
H14_14	Teréz	–	–	–	–	–
H14_15	Teréz	Octopine	–	–	–	–
H14_16	Teréz	Octopine	–	–	–	–

*Presence of opine in the opine assay is indicated by the name of opine type detected by the specific test while minus signs indicate the absence of opine. Plus and minus signs in the PCR assay column indicate the presence and absence, respectively, of PCR products corresponding to the *pehA* gene of *Allorhizobium vitis* (PGF–PGR) or *virD* gene of *A. tumefaciens* (VirD2A–VirD2E) in the extracted crown gall DNA.*

## Results

### A Modified Illumina V3–V4 16S rRNA Primer Reduces Co-amplification of Grapevine Plastid DNA

A preliminary analysis of three Hungarian CG samples based on amplicons generated from primers S-D-Bact-0341-b-S-17 and S-D-Bact-0785-a-A-21 showed that nearly all of the entire sequencing reads originated from grapevine chloroplast (80–90%) and mitochondrial (5–20%) rDNAs (Figure 1C), rendering large-scale amplicon sequencing of CG microbiota potentially cost-ineffective using the standard primer pairs. A new reverse primer with a single base mismatch to the grapevine chloroplast 16S ribosomal DNA at its 3′-end was subsequently designed (Figure 1A). Based on *in silico* analysis, the newly designed primer exhibited high coverage across various phyla with a notable reduction in a few bacterial phyla such as Chloroflexi, Synergistetes, Planctomycetes, and Cyanobacteria (Figure 1B). A near 100% reduction in the coverage for the phylum Cyanobacteria is consistent with the relatedness of the chloroplast to this phylum. Amplicon sequencing of the three previously sequenced Hungarian samples using the modified primer resulted in nearly zero recoveries of chloroplast-derived reads, indicating the efficiency of the new primer in inhibiting the co-amplification of highly abundant chloroplast 16S rRNA gene from the CG samples. Although the modified primer still co-amplified the host mitochondrial 18S rRNA gene due to the lack of 3′-end mismatch (Figure 1A), we observed sufficient relative abundance of bacterial reads across the three samples (30–90%) to justify its use in subsequent microbiota analysis (Figure 1C).

### A Small Crown Gall Core Microbiota

**FIGURE 2:**
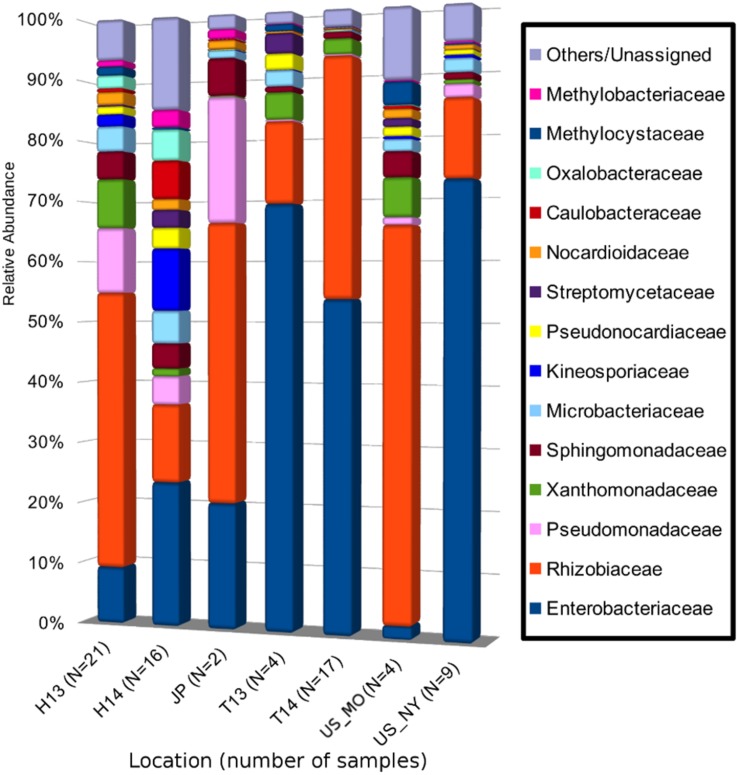
Mean relative abundance of bacterial 16S rRNA gene sequences at the family level of taxonomic classification in each sampling site. Each bacterial family is represented as a different color in the bar chart. The combined relative abundances total to 100% for each site. H13, Hungary (Heves) samples collected in 2013; H14, Hungary (Bacs-Kiskun) samples collected in 2014; T13, Tunisia (Regueb) samples collected in 2013; T14, Tunisia (Regueb) samples collected in 2013; US_MO, United States (MO) samples collected in 2013; US_NY, United States (NY) samples collected in 2014; JP, Japan (Okayama) samples collected in 2004 and 2013. Numbers in brackets indicate the number of crown gall samples collected from each site.

**FIGURE 3:**
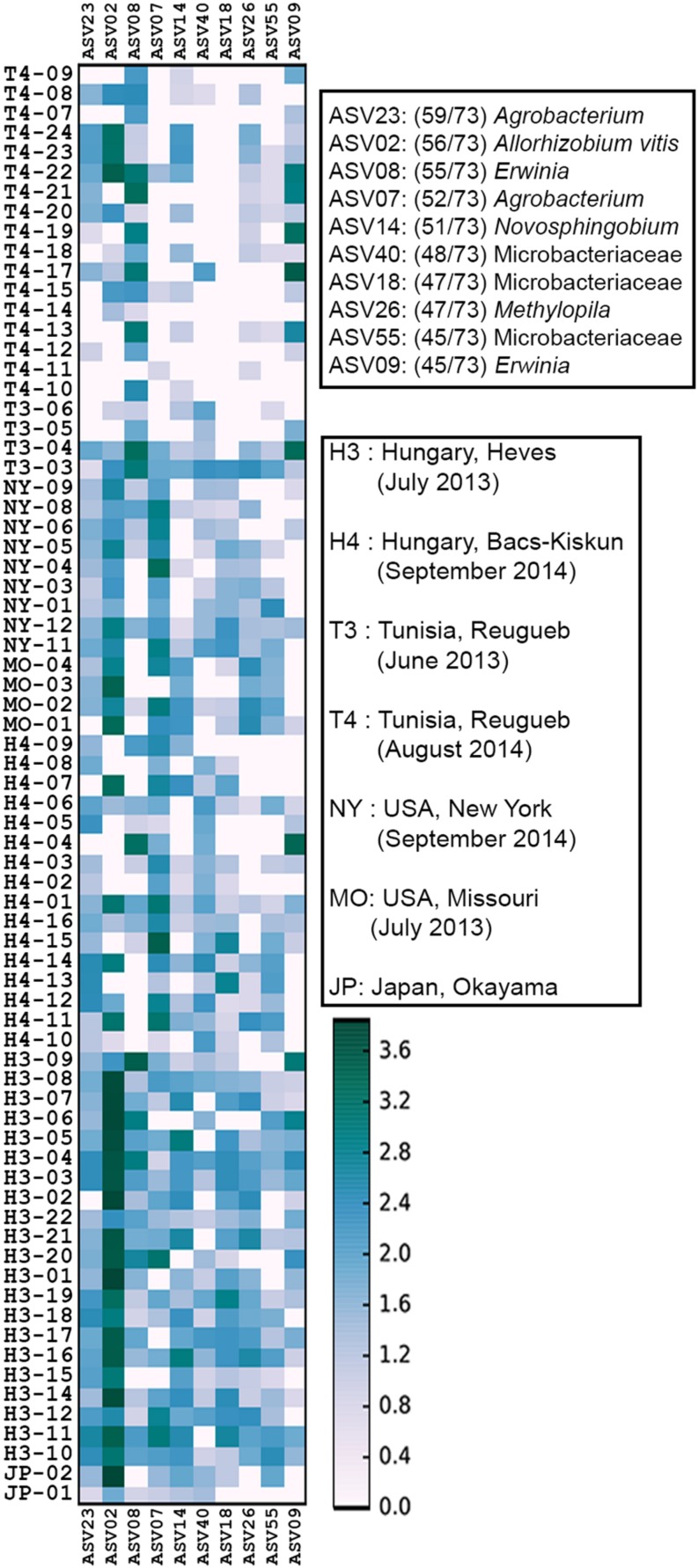
Heat map of core microbiome abundance across 73 crown gall samples. The *x*-axis shows all 10 core ASVs in the order of decreasing prevalence. The prevalence (numbers in brackets next to each ASV) and classification of the 10 core ASVs to the lowest possible taxonomic rank were shown in the upper right legend. The heatmap scale indicates the abundance of ASV (normalized to 10,000 reads/sample) in a 10-based logarithmic scale.

A total of 5,701 ASVs were generated from the de-replicated high quality overlapped paired-end reads of which 5,622 were inferred to be of bacterial origin (non-chloroplast and non-mitochondrial) (Supplementary Data S1 and Supplementary Table S2). Relative abundance calculation at the family level indicates that >80% of the reads from each collection site could be classified into 14 core microbial families (Figure 2 and Supplementary Table S3). The cumulative relative abundance of reads mapping to Rhizobiacea and Enterobacteriaceae is generally high across sites ranging from 35 to 90% (Figure 2). Some microbial families are only abundant at a particular site. For example, most reads assigned to Kineosporiaceae and Caulobacteraceae were found in samples collected from Hungary in September 2014. Ten ASVs were found to be present in >60% of the samples (Figure 3). ASV2 corresponding to *A. vitis* is the second most prevalent ASV with presence in >75% of the samples. This ASV is highly abundant in a majority of the Hungarian samples collected in July 2013 with an average 35% relative abundance (39% median relative abundance). On the contrary, the most prevalent ASV23 that was assigned to the genus *Agrobacterium* has a relative abundance of only <10% across all samples. Notably, the core microbiota matrix of most Tunisian samples is generally sparser with some of them even missing the prevalent ASV2 and ASV23 (Figure 3).

### Low Occurrence of *Allorhizoium vitis* in Some Opine-Producing Grapevine Crown Galls

Ten out of 16 Hungarian CGs collected at the beginning of fall 2014 (Supplementary Table S1) contained either octopine or vitopine (Table 1). Of the 10 opine-containing Hungarian CGs, only 3 (H14_1, H14_7, and H14_11) were positive for *A. vitis* when tested with *A. vitis*-specific primers consistent with the high relative abundance of ASV2 (13–19%) in these samples (Figure 3). However, sample H4_14 that has a slightly lower ASV2 abundance (9.5%) in addition to a few samples with <1% ASV2 relative abundance were reported as *A. vitis*-negative by the PCR approach. None of the samples were tested positive for *A. tumefaciens* that was occasionally identified as the causative agent of CG infection in grapevine (Table 1).

### Crown Gall Microbiota Is Variable Across Sites

**FIGURE 4:**
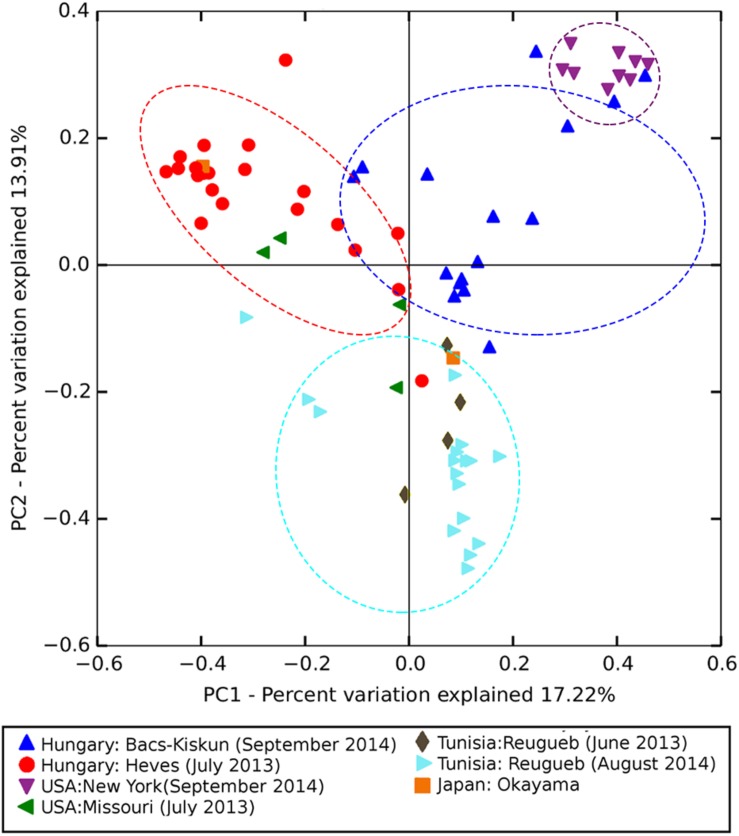
Principal coordinate analysis (PCoA) ordination based on Bray–Curtis dissimilarity matrix showing significantly different (ANOSIM global *R* = 0.63, *p-*value = 0.001) microbial composition among sampling sites. Points in the PCoA plot represent crown gall samples each colored and shaped according to the sampling site. Points enclosed by dotted lines represent samples from major sampling sites (*N* > 10 samples).

Analysis of similarities indicates that the microbial composition among sites is significantly different (ANOSIM global *R* = 0.63, *p-*value = 0.001). As expected, samples [represented by data points on the principal coordinate analysis (PCoA) plot] were broadly clustered based on their collection sites with some notable exceptions among the Hungarian samples collected in late September 2014 (H4 in Figure 4). Four of the H4 samples were positioned close to the New York cluster while two were observed in the upper left quadrant consisting mostly of 2013 samples from Hungary which represent the younger (approximately 2-month-old) CGs. On the other hand, the Tunisian samples were only found in the lower PCoA quadrants with a majority of them clustered along the vertical axis in the lower right quadrant. ASVs belonging to the genus *Pseudomonas* represent four out of five most significantly enriched ASVs among the 2013 samples from Hungary (Table 2). All five of the most significantly enriched ASVs in both New York and Tunisia sites were assigned to the family Enterobacteriaceae with one of them (ASV15) having an assignment at the genus level to the genus *Erwinia*. On the other hand, the differentially abundant ASVs in the 2014 samples from Hungary are more diverse, consisting of three Alphaproteobacteria, one Betaproteobacteria, and one Flavobacteria ASVs (Table 2).

**TABLE 2:** Top five significantly enriched ASVs by sampling sites.

**ASV**	**Taxonomy**	**Group 1^a^**	**ARA (%)**	**Group 2**	**ARA (%)**	***p-*value**
ASV198	p_Proteobacteria;c_Gammaproteobacteria;o_Pseudomonadales;f_Pseudomonadaceae;g_Pseudomonas;	H13	0.31416	Non-H13	0.0002	1.67*E*−65
ASV404	p_Proteobacteria;c_Gammaproteobacteria;o_Pseudomonadales;f_Pseudomonadaceae;g_Pseudomonas;	H13	0.0995	Non-H13	0.00001	3.50*E*−49
ASV101	p_Proteobacteria;c_Gammaproteobacteria;o_Pseudomonadales;f_Pseudomonadaceae;g_Pseudomonas;	H13	0.30347	Non-H13	0.00083	2.04*E*−40
ASV11	p_Proteobacteria;c_Gammaproteobacteria;o_Pseudomonadales;f_Pseudomonadaceae;g_Pseudomonas;	H13	4.72383	Non-H13	0.2748	1.33*E*−34
ASV539	p_Bacteroidetes;c_Sphingobacteriia;o_Sphingobacteriales;f_Sphingobacteriaceae;g_Pedobacter;	H13	0.01727	Non-H13	0.00034	1.88*E*−27
	
ASV277	p_Proteobacteria;c_Alphaproteobacteria;o_Caulobacterales;f_Caulobacteraceae;	H14	0.14546	Non-H14	0.00026	1.18*E*−102
ASV30	p_Bacteroidetes;c_Flavobacteriia;o_Flavobacteriales;f_Weeksellaceae;g_Chryseobacterium;	H14	1.02049	Non-H14	0.00026	2.94*E*−101
ASV181	p_Proteobacteria;c_Alphaproteobacteria;o_Sphingomonadales;f_Sphingomonadaceae;g_Novosphingobium;	H14	0.26148	Non-H14	0.00046	8.26*E*−99
ASV408	p_Proteobacteria;c_Alphaproteobacteria;o_Rhizobiales;f_Bradyrhizobiaceae;g_Bradyrhizobium;	H14	0.08687	Non-H14	0.00015	1.39*E*−82
ASV485	p_Proteobacteria;c_Betaproteobacteria;o_Burkholderiales;f_Burkholderiaceae;	H14	0.06765	Non-H14	0.00007	1.50*E*−73
	
ASV2917	p_Proteobacteria;c_Gammaproteobacteria;o_Enterobacteriales;f_Enterobacteriaceae;	NY	0.08737	Non-NY	0.0001	1.89*E*−61
ASV3878	p_Proteobacteria;c_Gammaproteobacteria;o_Enterobacteriales;f_Enterobacteriaceae;	NY	0.12115	Non-NY	0.00025	3.38*E*−58
ASV828	p_Proteobacteria;c_Gammaproteobacteria;o_Enterobacteriales;f_Enterobacteriaceae;	NY	0.1007	Non-NY	0.0002	1.41*E*−55
ASV1502	p_Proteobacteria;c_Gammaproteobacteria;o_Enterobacteriales;f_Enterobacteriaceae;	NY	0.02389	Non-NY	0.00011	2.45*E*−42
ASV1154	p_Proteobacteria;c_Gammaproteobacteria;o_Enterobacteriales;f_Enterobacteriaceae;	NY	0.02789	Non-NY	0.00009	1.68*E*−40
	
ASV15	p_Proteobacteria;c_Gammaproteobacteria;o_Enterobacteriales;f_Enterobacteriaceae;g_Erwinia;	Tunisia	3.0591	Non-Tunisia	0.00036	3.29*E*−113
ASV16	p_Proteobacteria;c_Gammaproteobacteria;o_Enterobacteriales;f_Enterobacteriaceae;	Tunisia	2.70482	Non-Tunisia	0.00044	1.06*E*−92
ASV27	p_Proteobacteria;c_Gammaproteobacteria;o_Enterobacteriales;f_Enterobacteriaceae;	Tunisia	2.04908	Non-Tunisia	0.00323	2.38*E*−71
ASV12	p_Proteobacteria;c_Gammaproteobacteria;o_Enterobacteriales;f_Enterobacteriaceae;	Tunisia	3.91837	Non-Tunisia	0.00238	1.52*E*−69
ASV226	p_Proteobacteria;c_Gammaproteobacteria;o_Enterobacteriales;f_Enterobacteriaceae;	Tunisia	0.29893	Non-Tunisia	0.00005	5.24*E*−67

*Group 1 is the reference group (samples from a specific sampling site) that will be compared against Group 2 (all other samples that are not from Group 1 sampling site). ARA, average relative abundance. ^*a*^H13, 2013 samples from Heves, Hungary (*n* = 20); H14, 2014 samples from Bacs-Kiskun, Hungary (*n* = 16), NY, 2013 samples from New York, United States (*n* = 9); Tunisia, 2013 and 2014 samples from Regueb, Tunisia (*n* = 21).*

### Associations Between Crown Gall Microbes

**FIGURE 5:**
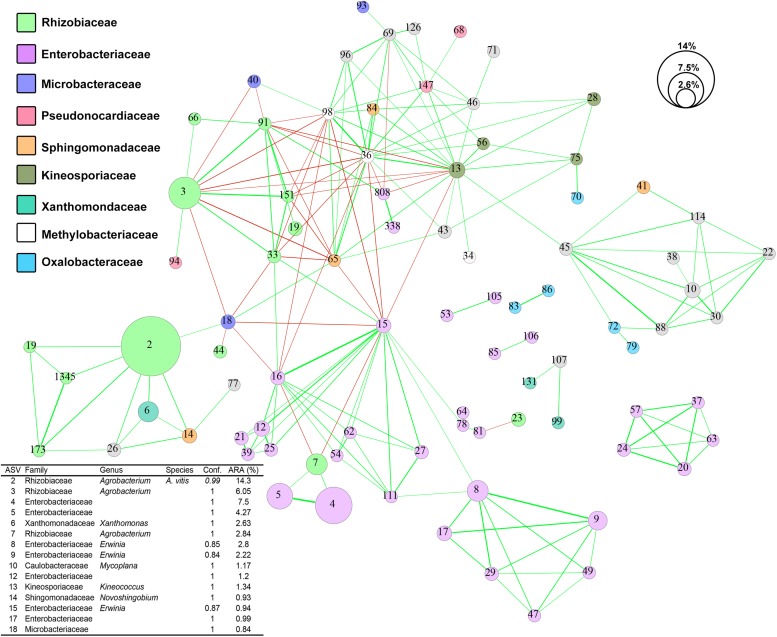
Co-occurrence network of ASV sequences from 73 crown gall samples. Strong (–0.5 < *R* < 0.5) and significant (*p-*value < 0.001) co-occurrences between ASVs are displayed by lines connecting the nodes. The line thickness reflects the strength of the correlation while the color reflects positive (green) or negative (red) associations. Each node represents one ASV and was colored based on RDP naïve Bayesian taxonomic classification to the family level. The size of each node reflects the average percentage relative abundance of the ASV they represent. Conf., confidence score of RDP taxonomic assignment; ARA, average relative abundance.

Using SparCC, we generated a microbial interaction network capturing 194 significant associations (red and green lines in Figure 5) among 86 ASVs (nodes in Figure 5) across the 73 CG samples. The 86 ASVs mostly belong to nine microbial families with nearly half of them assigned to the families Enterobacteriaceae and Rhizobiaceae (purple and green nodes in Figure 5). ASV2 (*A. vitis*), the most abundant ASV, showed positive co-occurrence relationships (*R* > 0.5 and *p-*value < 0.001) with three Rhizobiaceae ASVs (ASVs 19, 173, and 1345), one *Xanthomonas* ASV (ASV6), one *Novosphingobium* ASV (ASV14), one Methylocystaceae ASV (ASV26), and one Microbacteriaceae ASV (ASV18). On the other hand, ASV3, the second most abundant ASV assigned to the genus *Agrobacterium*, exhibits mixed co-abundance relationships with 11 ASVs which proceed to form a complex interaction network (Figure 5). Furthermore, three small network clusters consisting exclusively of Enterobacteriaceae ASVs were also observed and may represent site-specific microbial interactions.

### OTU Clustering Approach Underestimated *Agrobacterium* Diversity in CG Microbiota

**FIGURE 6:**
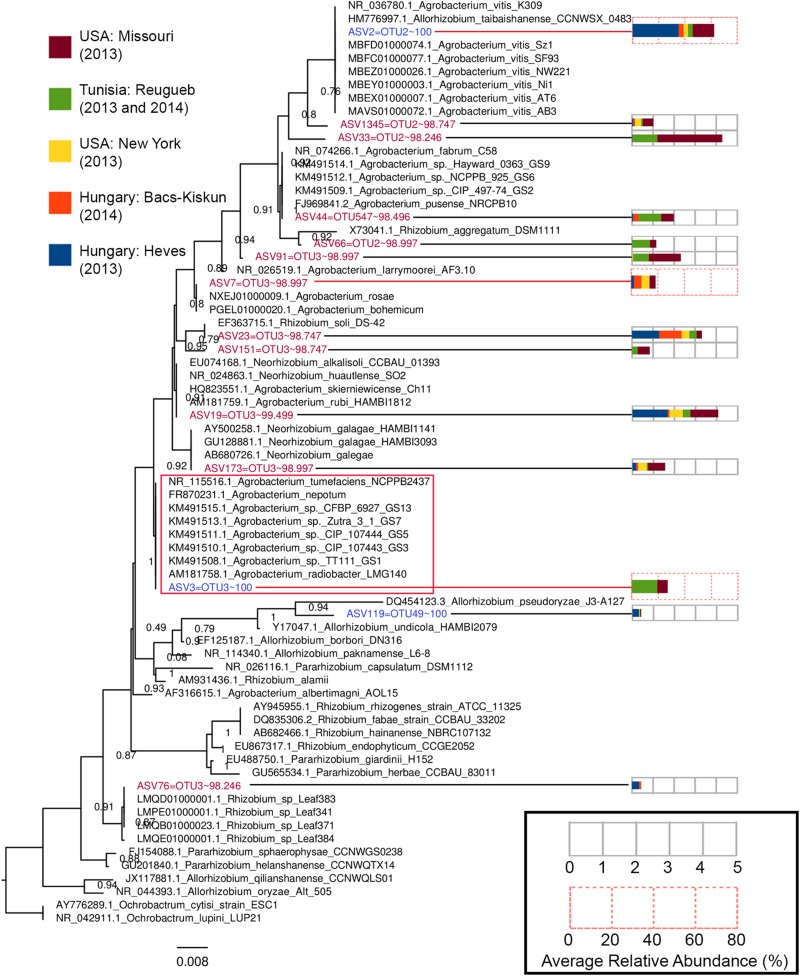
A maximum-likelihood tree constructed based on the alignment of *Agrobacterium* and *Allorhizobium* ASVs to the V3–V4 16S rRNA gene region of *Agrobacterium, Allorhizobium*, *Pararhizobium*, and *Rhizobium* strains. The tree was rooted with members from the genus *Ochrobactrum* as the outgroup. Tips representing ASVs included in the tree construction were colored based on the percentage nucleotide identity (values after the tilde symbols) of an ASV to its best matching OTU, with blue and red indicating perfect match (100% identity) and near-perfect (98% < *x* < 100% identity) match, respectively. Numbers at nodes indicate Shimodaira–Hasegawa (SH)-like local support values and branch lengths indicate the number of nucleotide substitutions per site. Codes preceding the genus name of each type strain are their NCBI accession numbers. The GS# labels present at the end of some *Agrobacterium* tip labels correspond their genospecies and the red box indicates an *Agrobacterium* clade consisting of multiple *Agrobacterium* genospecies with zero branch length or identical 16S rRNA V3–V4 gene sequence. Each ASV tip label associated with an abundance profile (right) that displays the average percentage relative abundance of the ASV in the major sampling sites.

Operational taxonomic unit clustering approach generated three OTUs with an exact match to ASV2, ASV3, and ASV119 (Figure 6 and Supplementary Data S2). The other 11 non-matching ASVs exhibits >97% nucleotide similarity to the three OTUs, suggesting that biological sequences corresponding to these ASVs would have been removed/clustered and represented by only a few OTUs. Despite exhibiting high sequence similarity to their corresponding OTUs, phylogenetic clustering showed that most of these 11 non-matching ASVs formed a tight cluster with known type strains, indicating that these are likely *bona fide* biological sequences (Figure 6). ASV7 is identical to two different *Agrobacterium* species, *A. rosae*, and *A. bohemicum*, underscoring inability of the V3–V4 region to resolve some *Agrobacterium* species. It is worth noting that despite being initially classified as *Agrobacterium* by RDP, some of these ASVs are more closely related to *Rhizobium* (ASV76, ASV23, and ASV151) and *Neorhizobium* (ASV173). Although ASV33 and ASV1345 do not show identical match to any type strain, it is unlikely that they arose due to sequencing artifact given their relative abundance among samples collected from two distant North American sites (Figure 6).

## Discussion

To define the microbiota of CG across, we sampled 73 CGs from various grape cultivars located in seven vineyards across four continents of the Northern Hemisphere. In contrast to the work done by Faist et al. (2016) who limited sampling to a single vineyard, our study provides a more comprehensive insight into the CG microbiota. Chloroplast contamination was dramatically reduced in this study using a modified reverse primer, albeit at the expense of reduced primer coverage for a few phyla which are not commonly plant associated (Hirsch and Mauchline, 2012; Turner et al., 2013; Schlaeppi and Bulgarelli, 2015). Similarly leveraging on primer mismatch to host plastid genome, a recent study has recommended the use of standard and unmodified primer pair “799F-1391R” targeting the V5–V7 region that was shown to dramatically reduce co-amplification of poplar plastid (Beckers et al., 2016). Given that a majority of Illumina-based microbiota studies target the V3, V4, or V3–V4 region (Klindworth et al., 2013; Gilbert et al., 2014a), protocol modifications directly aimed at overcoming co-amplification of host DNA may be beneficial. An attractive and potentially more cost-effective approach for future large-scale studies would be to design blocking primers that are complementary to the host chloroplast and mitochondrial rRNA gene (Hanshew et al., 2013; Beckers et al., 2016). A blocking primer will contain C3 spacer at its 3′-end that prevents extension during PCR when included into the standard PCR mix at an equal or higher concentration than the standard 16S primers (Arenz et al., 2015).

It is also worth noting that a majority of the sampling in this global study was performed between June and September, a critical period during which glucose concentration in the grape is most variable (Bauer et al., 1994). This may explain the variability in the microbiota composition across sample sites. It is also possible that the use of different DNA extraction methods may have contributed to the difference observed. However, given that the sampling was performed at multiple distant geographic regions at different time, the effect of such spatial and temporal variations on microbiota composition should outweigh the effect of variance in the DNA extraction method employed. Although examining the effect of extraction methodology on CG microbiota composition is beyond the scope of this study, similar studies have been conducted on non-CG samples with the general consensus that inter-sample variation always outweighed the variation in extraction method (Wesolowska-Andersen et al., 2014; Sinha et al., 2017).

The colony forming unit (CFU) of *A. vitis* from CG tumors of Riesling and Müller-Thurgau grapevine cultivars in Germany was previously shown to decrease dramatically after the month of June (Faist et al., 2016). Similarly, *A. vitis* was largely absent among Hungarian samples sampled in September 2014 but not July 2013 based on *A. vitis* PCR detection and amplicon sequencing assays. The absence of *A. vitis* in a majority of the opine-containing Hungarian CG samples suggests while opine serves as an attractant to *A. vitis*, there are additional biotic and/or abiotic factor(s) that can influence the population dynamics of *A. vitis* in CG tumors (Bhattacharya et al., 2010). On the other hand, high ASV3 to ASV2 ratio, indicating a strong dominance of *Agrobacterium* spp. over *A. vitis* was observed only in the Tunisian CGs. *Agrobacterium* spp. members were previously shown to be dominant in Tunisian soils based on culture-based method, an observation that was hypothesized to be due to the climatic and soil conditions of the country (Bouri et al., 2016). The diversity of *Agrobacterium* populations in Tunisian vineyards seems to be restricted to the genomic species G4, G7, and G9. Since *Agrobacterium* genomic species G9 was represented by ASV44 instead of ASV3, 16S rRNA reads mapping to ASV3 in the Tunisian CG tumors may originate from *Agrobacterium* spp. belonging to genomic species GS4 and/or GS7. The lack of Tunisian tumorigenic strains associated with genomic species G7 as determined by PCR-based detection of Ti-plasmid (VCF3-VCR3 primers; *virC*) lends support toward the affiliation of ASV3 in Tunisian CG tumors to *Agrobacterium* spp. genomic species G4 (Bouri et al., 2016). It is important to note that such a correlation cannot be applied to non-Tunisian samples that are lacking background microbial genetic data.

In addition to *A. vitis* (OTU_0003), Faist et al. (2016) also identified two additional OTUs (OTU_0005, *Pseudomonas* sp.; OTU_0008, *Enterobacter* sp.) showing high abundance and prevalence in tissues sampled from vines containing CG tumors during spring and autumn. Although our study did indeed demonstrate the prevalence and substantial abundance of *A. vitis*-linked ASV in a majority of the CG tumor tissue samples, no ASVs corresponding to *Pseudomonas* could be found in the core microbiota. However, we do observe a significant enrichment of *Pseudomonas*-linked ASVs in the Hungarian 2013 CG tissue samples, which may suggest the positive association of members from this genus during early CG formation possibly due to the ability to utilize opine compounds as a growth substrate (Bell et al., 1990). For example, the genome of *P. kilonensis* strain 1855-344, a strain which can catabolize octopine, has recently been shown to contain an octopine-catabolic operon named *ooxAB* (Eng et al., 2015). Interestingly, despite being present in the core microbiota, none of the Enterobacteriaceae-linked ASVs formed a significant co-occurrence with *A. vitis* (ASV2). However, several Enterobacteriaceae-linked ASVs were shown to be differentially abundant among samples from New York and Tunisia. The occurrence of members of the Enterobacteriaceae has recently been associated with the native microbiota of viticultural regions, and has been proposed to impart distinct chemical composition and sensory characteristics of regional wines (Bokulich et al., 2016). It has been suggested that plant-associated members of Enterobacteriaceae also play a part in the complex interactions among the environmental, temporal, plant-genetics, human, and other factors which influence grapevine growth and development collectively referred as “terroir” (Fischer et al., 1999; López-Rituerto et al., 2012). Within the family Enterobacteriaceae, members from the genera *Pantoea* and *Erwinia* are common plant inhabitants. However, the evolutionary relationships of members from these two genera could not be confidently established using the nucleotide sequence of the 16S rRNA gene alone. This uncertainty may be responsible for our failure to assign several Enterobacteriaceae-linked ASVs to the genus level. Since *Erwinia* and *Pantoea* spp. are readily culturable on agar medium, it may be more appropriate to infer their roles in the CG microbiota using a culture-based approach followed by biochemical characterization, whole genome sequencing, and comparative genomics (Hong et al., 2012; Gan et al., 2014; Walterson and Stavrinides, 2015).

The strong co-occurrence of *Xanthomonas* and *Novosphingobium* with *A. vitis* as observed in this study is consistent with the affiliation of their members with grapevine and more specifically with CG tumor tissues. For example, an AHL-producing *Novosphingobium* sp. strain Rr 2-17 was isolated from a CG tumor and its quorum sensing AHL-signal production was shown via transposon mutagenesis to be regulated by the stringent response gene, *rsh* (Gan et al., 2009). The TraR protein associated with conjugal transfer of Ti plasmid reacts strongly to the AHL signals generated by strain Rr 2-17, suggesting that it may play a role in amplifying the quorum sensing signal required to disseminate Ti plasmid (via conjugal plasmid transfer) among *Agrobacterium, Allorhizobium*, and potentially additional compatible strains in the tumor environments (Zhu and Winans, 2001; Zhang et al., 2002; Lowe et al., 2009). *In silico* identification of quorum sensing synthase gene from *Novosphingobium* genomes indicates most members exhibit the genomic potential to produce AHL (Gan et al., 2013, 2015, 2016). Known for their ability to mineralize complex aromatic compounds, it is possible that *Novosphingobium* spp. might harbor homologous genes associated with the catabolism of opine compounds or intermediates which have a structural resemblance to oxygenase-cleaved aromatic compound (Wilcke, 2000; Gan et al., 2011, 2012b; Mallick et al., 2011). *Xanthomonas* spp. also have been reported to be commonly present on grapevine leaves and bark, but absent from grapes or grapevine-associated soils as determined through culture-dependent and culture-independent approaches (Martins et al., 2013). The strong co-occurrence of *Xanthomonas* with *A. vitis* in CG tumors can be explained by the presence of high concentrations of lignin, cellulose, *N*-glycosylated proteins, and other cell wall precursors as well as cell wall degradation products found in bark on developing CG tumors (Blanvillain et al., 2007; Boulanger et al., 2014).

The identification and delineation of agrobacteria even based on full-length 16S rRNA gene sequences has been problematic as indicated by polyphyletic clustering pattern and low bootstrap support values, as observed in various phylogenetic trees even with high taxon sampling. The lack of informative sites is presumably due to the slower evolutionary rate of 16S rRNA among agrobacteria (Costechareyre et al., 2010). Despite being able to resolve amplicon down to the single base resolution, the monophyletic clustering of ASV2 with multiple genomic species of *Agrobacterium* exhibiting diverse and distinct ecological niches strongly suggests that caution needs to be exercised when inferring ecological interactions from 16S rRNA dataset. An improved understanding of the microbial interactions within such an environment can be gained through whole metagenome shotgun sequencing approach coupled with the ProxiMeta^TM^ Hi-C metagenome deconvolution method that can link plasmids to their hosts (Press et al., 2017). However, the challenges associated with strong host (grapevine) gDNA contamination will need to be addressed through increased sequencing depth and/or the selective removal of host methylated gDNA (Feehery et al., 2013).

## Conclusion

The widely used Illumina standard V3–V4 16S rRNA primers are not suitable for grapevine microbiota studies as they exhibit perfect match to the grapevine plastid 16S rRNA gene. We report a new pair of V3–V4 16S ribosomal RNA gene PCR primers which prevent the co-amplification of this gene region and used this primer pair to investigate the microbial community of 73 CG tissue samples collected from multiple distinct geographic regions. The CG microbial community is diverse and varies significantly across samples and vineyards.

## Data Availability

Sequencing data generated from this study have been deposited into the NCBI Sequence Read Archive under the BioProject code PRJNA490446.

## Author Contributions

HG, MS, and ES conceived the project, designed the experiments, and drafted the manuscript. ES, RF, SC, LK, AK, and TB collected the samples and performed the DNA extraction. HG performed the amplicon sequencing and conducted the bioinformatic analysis. All authors edited and contributed to the manuscript.

## Conflict of Interest Statement

The authors declare that the research was conducted in the absence of any commercial or financial relationships that could be construed as a potential conflict of interest.

## Supplementary Material

TABLE S1 Detailed metadata of crown gall tumors collected from different sites.

TABLE S2 Raw count data of bacterial ASVs and their taxonomic classification.

TABLE S3 Relative abundance (%) of bacterial ASVs and their taxonomic classification.

DATA S1 DNA sequences of unfiltered ASVs in fasta format.

DATA S2 DNA sequences of unfiltered OTUs in fasta format.
